# USP14 Inhibition Regulates Tumorigenesis by Inducing Autophagy in Lung Cancer In Vitro

**DOI:** 10.3390/ijms20215300

**Published:** 2019-10-24

**Authors:** Kyung Ho Han, Minseok Kwak, Tae Hyeong Lee, Min-soo Park, In-ho Jeong, Min Ji Kim, Jun-O Jin, Peter Chang-Whan Lee

**Affiliations:** 1 Department of Biomedical Sciences, University of Ulsan College of Medicine, Asan Medical Center, Seoul 05505, Korea; Kyungho1.han@gmail.com (K.H.H.); T.hyeong.lee@gmail.com (T.H.L.); harimaho88@gmail.com (M.-s.P.); D4inno@naver.com (I.-h.J.); mendi_@naver.com (M.J.K.); 2 Department of Chemistry, Pukyong National University, Busan 48513, Korea; mkwak@pukyong.ac.kr; 3 Department of Medical Biotechnology, Yeungnam University, Gyeongsan 38541, Korea

**Keywords:** ubiquitin-specific protease 14, deubiquitinating enzyme, lung cancer, autophagy

## Abstract

The ubiquitin–proteasome system is an essential regulator of several cellular pathways involving oncogenes. Deubiquitination negatively regulates target proteins or substrates linked to both hereditary and sporadic forms of cancer. The deubiquitinating enzyme ubiquitin-specific protease 14 (USP14) is associated with proteasomes where it trims the ubiquitin chain on the substrate. Here, we found that USP14 is highly expressed in patients with lung cancer. We also demonstrated that USP14 inhibitors (IU1-47 and siRNA-USP14) significantly decreased cell proliferation, migration, and invasion in lung cancer. Remarkably, we found that USP14 negatively regulates lung tumorigenesis not only through apoptosis but also through the autophagy pathway. Our findings suggest that USP14 plays a crucial role in lung tumorigenesis and that USP14 inhibitors are potent drugs in lung cancer treatment.

## 1. Introduction

Among the various cancers, lung cancer has the highest associated mortality worldwide. Owing to late-stage detection and poor treatment, patients with lung cancer have a survival rate lower (by approximately 15%) than those of other cancers. Non-small-cell lung cancer (NSCLC) is the most common type of lung cancer (approximately 85%) [1,2,3].

The ubiquitin–proteasome system (UPS) is well known as an essential regulator of several cellular pathways involving oncogenes. Deubiquitination negatively regulates target proteins or substrates in UPS. Deubiquitinating enzymes (DUBs) eliminate ubiquitin from functional proteins and scavenge ubiquitin during ubiquitination [4]. Ubiquitin-specific protease 14 (USP14) is one of the 100 DUBs and belongs to the mammalian 19S regulatory subunit. USP14 is abundantly present in most cancers and mediates proteasome activity in cancer cells [5,6]. Previous studies have shown that USP14 is highly expressed in patients with NSCLC, and it is related to tumor proliferation [7,8].

UPS and autophagy are the principal intracellular mechanisms controlling protein degradation, damage, and misfolding in eukaryotes. UPS is primarily responsible for the degradation of short-lived proteins under normal conditions. Contrarily, autophagy is generally responsible for cell homeostasis, cell differentiation, and cell death under starvation or stressful environments. Autophagic cells markedly induce cell death to escape from excessive stress. [9,10]. Recent studies have shown that USP14 regulates protein turnover via UPS and autophagy for proteome homeostasis [11,12,13,14]. Moreover, the inhibition of USP14 modulates proteasome activity and promotes mitophagy [15]. However, the role of USP14 in cancer cell death has not yet been clarified.

In this study, we aimed to study the expression of USP14 in patients with lung cancer and to demonstrate the role of USP14 inhibition and its underlying mechanism in lung tumorigenesis.

## 2. Results

### 2.1. USP14 Expression Level in Lung Cancer Tissues

**Figure 1:**
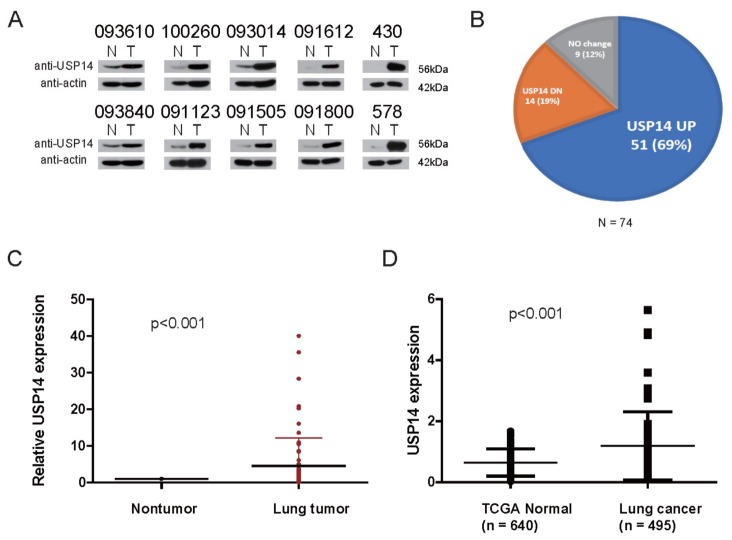
USP14 expression level in lung cancer tissues. (**A**) Representative immunoblot for USP14 and β-actin in 74 paired lung cancer tissues and their adjacent nontumor lung tissues (N, nontumorous, T, cancer tissue), (**B**) pie chart of USP14 expression using immunoblot analysis in 74 paired lung cancer tissues and nontumor tissues. β-Actin expression was used as an internal control, (**C**) USP14 expression levels in 74 lung cancer cases. *P*-values represent probabilities for USP14 expression levels between variable subgroups determined with two-sided Fischer’s exact test (tumor node metastasis), (**D**) expression levels of the USP14 gene in healthy individuals (Normal, *n* = 640) and patients with lung cancer (Tumor, *n* = 495) from the TCGA LUAD database. The box plot represents the mean expression of USP14 (±SD), and the horizontal line indicates the median value.

To identify the expression of USP14 in lung cancer tissues, we examined the protein levels of USP14 in 74 paired patient-derived lung cancer tissues and adjacent nontumor nontumor lung tissues using Western blotting with an USP14 antibody (Figure 1A and Supplementary Figure S1). We found that USP14 is expressed at high levels (68.9%, 51/74) in lung cancer tissues (Figure 1B), and its level was significantly different between the nontumor lung tissues and lung cancer tissues (Figure 1C). These findings were consistent with those of a previous study [7]. We also demonstrated that as per the TCGA Lung Adeno Carcinoma database, USP14 levels are higher in patients with lung cancer than in normal individuals, and this finding is consistent with our Western blotting data (Figure 1D). These findings indicate that USP14 overexpression is strongly correlated with tumorigenesis in lung cancer.

### 2.2. Inhibition of USP14 Decreases Tumor Proliferation and Invasion with IU1-47

**Figure 2:**
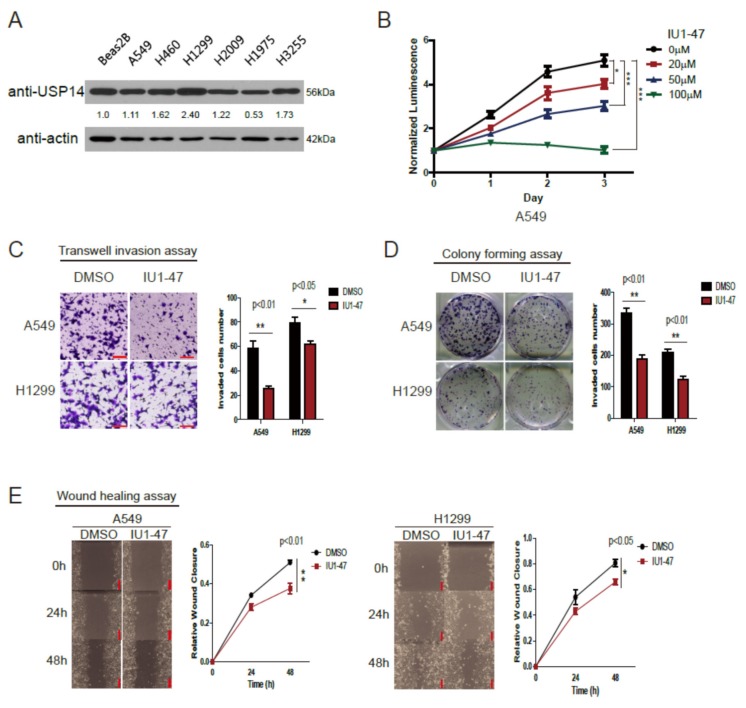
USP14 inhibition using IU1-47 decreases tumor growth. (**A**) USP14 expression levels in different lung cancer cells and normal cells, (**B**) USP14 inhibition using IU1-47 decreases cell viability in a dose-dependent manner (*n* = 5) (* *p* < 0.05, ** *p* < 0.01, *** *p* < 0.001), (**C**) A549 and H1299 cells treated with the USP14 inhibitor IU1-47. Transwell invasion was assessed after 24 h of incubation. Values represent the mean ± SD of the number of invaded cells (*n* = 5). Scale bar = 100 μm, (**D**) effect of USP14 inhibition on colony-forming (long-term cell proliferation) potential of A549 and H1299 cells (*n* = 3). A549 and H1299 cells treated with IU1-47 or DMSO were cultured for 10 days, and the colonies were stained with crystal violet and counted, (**E**) effect of USP14 inhibition on A549 and H1299 cells. Wound closure was determined at 24 and 48 h. Data in the graph are shown as mean ± SD compared with the data of control cells (*n* = 8).

To demonstrate the role of USP14 in tumorigenesis, we first examined endogenous USP14 protein in lung cancer cell lines. Endogenous USP14 protein levels were higher in the lung cancer cell lines A549, H460, H1299, H2009, and H3255 than in the normal lung cell line Beas2B but not in the lung cancer cell line H1975 (Figure 2A). A549 and H1299 cells were used for further experiments. We investigated whether USP14 inhibition suppresses tumor cell proliferation using the USP14 inhibitor IU1-47. Compared with the dimethyl sulfoxide-treated control cells, the IU1-47-treated cells exhibited decreased proliferation in a dose-dependent manner (Figure 2B). We also performed transwell invasion, colony formation, and wound healing assays using A549 and H1299 cells as well as IU1-47 (20 µM). These results demonstrated that the USP14 inhibitor-treated cells had significantly decreased proliferation, migration, and invasion compared with the control cells (Figure 2C–E). These findings suggest that USP14 is positively related to cell proliferation, migration, and invasion within lung cancer.

### 2.3. USP14 Knockdown with USP14 Small Interfering RNA (siRNA) Suppresses Tumor Proliferation and Invasion

**Figure 3:**
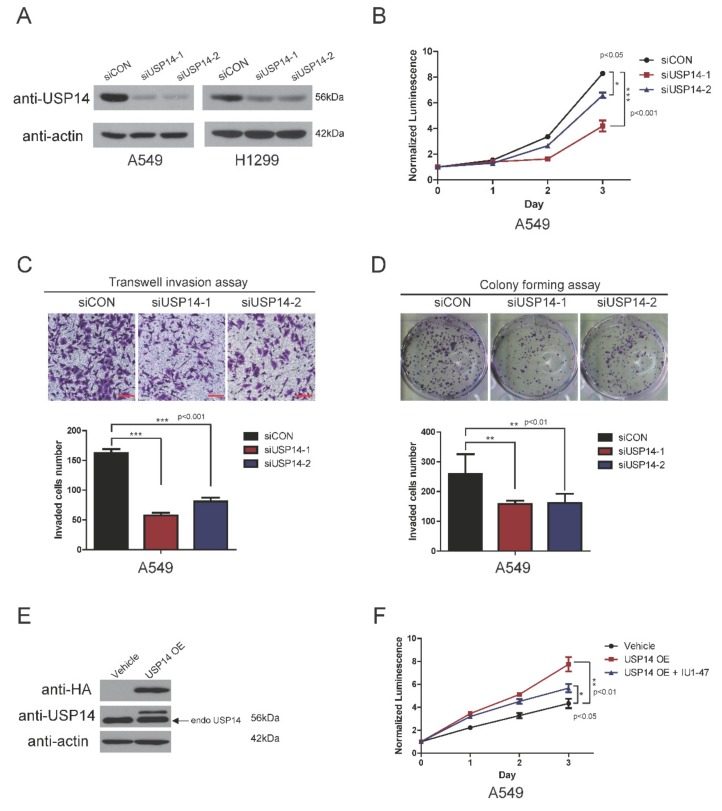
USP14 inhibition using siRNA in lung cancer cells. (**A**) Immunoblot analysis for USP14 using siRNA in A549 and H1299 cells. (**B**) Effect of USP14 knockdown on cell viability (*n* = 5). (**C**) Effect of USP14 knockdown on cell migration measured using the transwell invasion assay. Invasion was assessed after 24 h of incubation. Values are expressed as mean ± SD of the number of invaded cells (*n* = 5). Scale bar = 100 μm. (**D**) Effect of USP14 knockdown on colony-forming (long-term cell proliferation) potential of A549 cells (*n* = 3), (**E**) immunoblot of USP14 in A549 cells that are stably overexpressing USP14, (**F**) effect of USP14 overexpression on the cell viability of A549 cells. Cell viability was monitored using a CellTiter-Glo luminescent assay (with normalization to control wild-type cells at day 0 (*n* = 5)).

To confirm whether using IU1-47 decreases cell proliferation, invasion, and colony formation in lung cancer, we tested USP14 knockdown using siRNA on A549 and H1299 cells. A549 and H1299 cells were transfected with two USP14 siRNA and one control. USP14 expression decreased in A549, H1299, and H1975 cells (Figure 3A and Supplementary Figure S2). USP14 knockdown using siRNA was consistent with the finding of a previous study using IU1-47 and our own results described above [8]. USP14 knockdown using siRNA decreased cell proliferation, invasion, and colony formation (Figure 3B–D and Supplementary Figure S2). In contrast to USP14 knockdown, USP14 was stably overexpressed in A549 cells (Figure 3E). The results showed that USP14 overexpression markedly increases cell proliferation compared with vehicle or IU1-47-treated cells (Figure 3F). Taken together, these results confirmed that USP14 was involved in lung tumorigenesis.

### 2.4. USP14 Inhibition Does Not Induce Apoptosis but Induces LC3 Autophagic Cell Death

**Figure 4:**
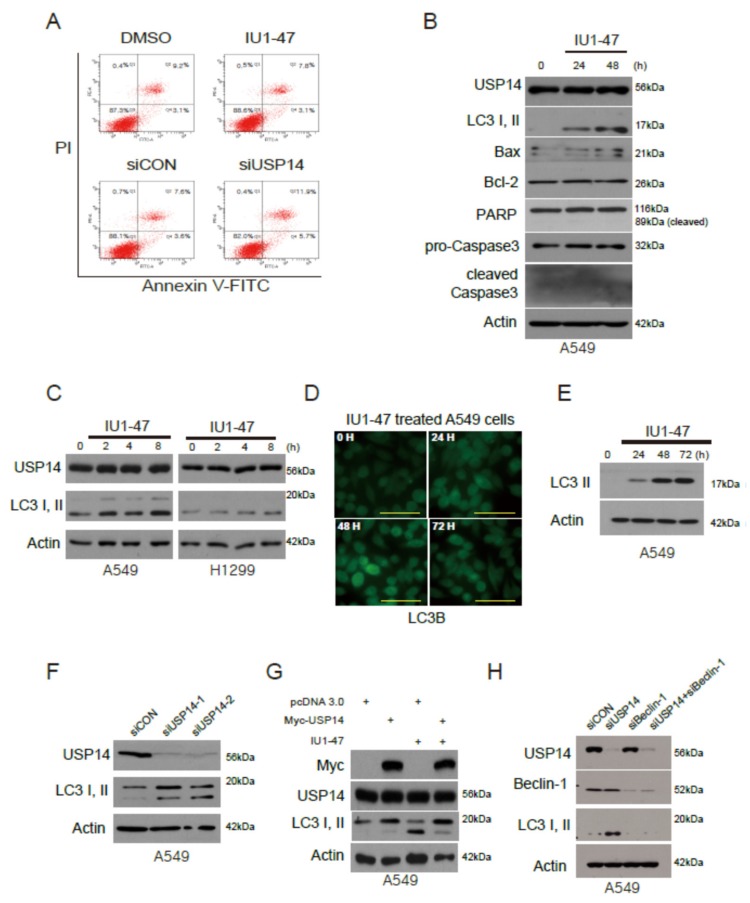
USP14 inhibition induces LC3 lipidation. (**A**) USP14 inhibitor (IU1-47)- and siUSP14-treated A549 cells tested using the annexin-V apoptosis assay. (**B**) A549 cells were treated with the USP14 inhibitor IU1-47 and then lysed and immunoblotted with anti-USP14, autophagy proteins (LC3 I and II), apoptotic cell death-associated proteins (Bax, Bcl-2, pro-caspase3, and cleaved caspase3), and β-actin antibodies. (**C**) A549 and H1299 cells were treated with IU1-47 and immunoblotted with anti-USP14 and LC3 I and II antibodies. (**D**–**E**) A549 cells were transiently transfected with GFP-LC3B/treated with IU1-47. Fluorescence and Western blotting data were collected for 3 days. Scale bar = 50 μm. (**F**) A549 cells were treated by USP14 knockdown and immunoblotted with anti-USP14 as well as LC3 I and II antibodies. (**G**) Lysates from A549 cells were treated with USP14 transfection or IU1-47 were immunoblotted with anti-USP14 beclin-1, LC3 I and II, and β-actin antibodies. (**H**) A549 cells were treated by USP14 knockdown or beclin-1 knockdown and immunoblotted with anti-USP14 beclin-1, LC3 I and II, and β-actin antibodies.

USP14 inhibition was found to strongly suppress cell proliferation in lung cancer. To demonstrate the cell death mechanism with USP14 protein, we detected apoptotic cells stained positive for annexin-V in A549 cells. FACS analysis showed that the IU1-47- and siRNA-treated cells did not induce apoptosis (Figure 4A). Next, we examined the expression of apoptotic and autophagic proteins under IU1-47 treatment in A549 cells. Remarkably, USP14 inhibition induced cell death not via the apoptotic proteins Bax, Bcl-2, pro-caspase3, and cleaved caspase3, but via the autophagy protein LC3 II (Figure 4B and Supplementary Figure S3A,B). The level of the autophagy protein LC3 I or II increased with IU1-47 or USP14 siRNA in A549 and H1299 cells (Figure 4C,F). To investigate whether the USP14 inhibitor induces autophagy in vitro, we used GFP-LC3B or LC3B antibody with USP14 inhibitor in A549 cells. We observed the autophagosomes of GFP-LC3 puncta and LC3 II protein. The level of LC3 proteins significantly increased in USP14 inhibitor-treated cells (Figure 4D–E).

When USP14 was transfected and treated with IU1-47 in A549 cells, we observed that the conversion of soluble LC3 I to membrane-bound LC3 II was elevated by USP14 inhibition. However, LC3 I and II levels were reversed by USP14 (Figure 4G). We further investigated whether beclin-1, a key upstream autophagic protein, regulated the LC3 II protein level. LC3 II protein levels did not markedly change with a beclin-1 knockdown in A549 cells (Figure 4H and Supplementary Figure S3C). Taken together, these findings suggest that USP14 is involved in the autophagy pathway.

## 3. Discussion

In this study, we report that the deubiquitinating enzyme USP14 is highly overexpressed in human lung cancer and USP14 inhibition suppresses cell proliferation, migration, and invasion in lung cancer. Conversely, USP14 overexpression in lung cancer cells markedly increases cell proliferation, migration, and invasion. Remarkably, USP14 regulated cell proliferation in lung cancer not by apoptosis but by autophagy.

A previous study has reported that USP14 inhibition using shRNA downregulates tumor cell proliferation [8]. Thus, we used IU1-47, which is a new small-molecule inhibitor of USP14 in lung cancer cell lines. IU1-47 is a 10-fold more potent USP14 inhibitor than IU1 [16]. Our results corroborate those of a previous study wherein USP14 downregulation decreased tumor growth [8]. We observed similar results with inhibition of USP14 using IU1-47 in lung cancer cell lines.

Cancer cell death involves four major cellular mechanisms: apoptosis, autophagy, necrosis, and necroptosis [17,18,19,20,21]. To successfully eliminate or suppress tumor cells, all these mechanisms must operate, and interact together, but also play independent roles in the human body. We have demonstrated here that autophagy-induced cell death is involved with USP14 in lung cancer. Previous studies have shown that USP14 regulates autophagy by interfering with beclin-1 [11,13,15,22]. For example, in breast carcinoma cells, it negatively controls tumor progression by blocking autophagy induction of beclin-1. We also found that USP14 inhibition significantly increases LC3 II expression and suppresses lung cancer progression. Furthermore, LC3 II was not induced when beclin-1 or USP14 was silenced by siRNA and inhibitors. Moreover, it has been shown that USP14 regulates Wnt/β-catenin signaling pathway by regulating Deshevelled (Dv1) protein in colon cancer [23]. In lung cancer, we did not see any regulation of Dv1 except LC3 II expression (data not shown). These findings indicate that USP14 is more strongly involved in autophagy than in apoptosis in lung cancer.

Overall, our results demonstrated that USP14 is upregulated in lung cancer and that it could be suppressed by IU1-47, an USP14 inhibitor. Importantly, USP14 is not involved in apoptosis but is involved in autophagy. These findings suggest that USP14 is a promising target in lung cancer therapy.

In conclusion, USP14 regulates lung tumorigenesis via autophagy. However, it remains unclear how USP14 contributes to tumor cell death and the autophagy pathway. Therefore, further studies are required to completely understand the functional role of USP14 in lung cancer. Finally, our research suggests that USP14 inhibitor can be used as a target in lung cancer therapy.

## 4. Materials and Methods

### 4.1. Patients and Lung Tissue Samples

Experiments were performed after obtaining informed consent from patients and approval from the Institutional Review Board of the Asan Medical Center and the University of Ulsan College of Medicine (2014-0960, date of approval: 5 July 2017). Human lung tissue samples were obtained from the Asan Bio Resource Center [2014-20(89)].

### 4.2. Cell Culture

A549 and H1299 cell lines were obtained from American Type Culture Collection. The cells were cultured in Dulbecco’s modified Eagle’s medium (DMEM, HyClone, GE Healthcare, Chicago, IL, USA) or RPMI-1640 medium (HyClone) supplemented with 10% fetal bovine serum (FBS, Corning Inc., Corning, NY, USA), and 1% penicillin–streptomycin (Gibco, Invitrogen, Carlsbad, CA, USA) and then incubated in a 5% CO_2_ incubator at 37 °C.

### 4.3. Immunoblotting

Human lung tissue samples and cells were rapidly homogenized in 1% sodium dodecyl sulfate (SDS) lysis buffer (40 mM Tris-Cl pH 8.0, 150 mM NaCl, and 1% SDS) using a 23G syringe. Then, the lysates were collected and quantified using the BCA protein assay kit (Pierce, ThermoFisher, Rockford, IL, USA), USP14 (Novus, Centennial, CO, USA), LC3 (Novus), Beclin-1 (Cell Signaling, Danvers, MA, USA), Bax (BD biosciences, San Jose, CA, USA), Bcl-2 (Santa Cruz, Dallas, TX, USA), Caspase-3 (Cell signaling), cleaved Caspase-3 (Cell Signaling), HA (Covance, Biolegend, San Diego, CA, USA), and β-actin (Santa Cruz) were analyzed by Western blotting using a secondary antibody (Thermo, Rockford, IL, USA) detected via enhanced chemiluminescence (Bio-Rad, Hercules, CA, USA).

### 4.4. Cancer Genome Atlas (TCGA) Analysis

Gene expression data of RNAseq2 level 3 provided in TCGA were analyzed to compare USP14 expression levels between nontumor and lung cancer tissues. Normal data were obtained from 21 tumor-adjacent nontumor lung tissues including lung cancer-matched nontumor tissues. The expression values of USP14 were transformed to log2 fold-change at the level normalized by the RSEM software (GNU general public license, https://deweylab.github.io/RSEM).

### 4.5. Transfection and siRNA

Cells were transfected with a plasmid using Lipofectamine 3000 (Invitrogen, Carlsbad, CA, USA) or iNfect (Intron, Seoungnam, South Korea) according to the manufacturer’s specifications. The transfected cells were harvested after 48 h for analysis. Protein cell lines showing stable expression were generated using a retroviral vector and were selected with Puromycin (Invitrogen). Lipofectamine RNAiMax (Invitrogen) was used for knockdown with siRNA according to the manufacturer’s specifications.

### 4.6. Cell Viability, Wound Healing, Colony Formation, and Invasion Assays

To assess cell viability, the cells were seeded at a density of 2 × 10^4^ cells/well in a 96-well cell culture plate and analyzed on days 0–3. At each point of analysis, the CellTiter-Glo reagent (Promega, Madison, WI, USA) was added and the plate was incubated for 5 min on a shaker. For the wound healing assay, cells were seeded at a density of 6 × 10^5^ cells/well in a 6-well cell culture plate. Then, the cells were wounded with a 200 μL pipette tip, washed twice with PBS, and analyzed on days 0–3 under a microscope. For the colony formation assay, the cells were seeded at a density of 1 × 10^3^ cells/well in a 6-well cell culture plate and incubated for 1–2 weeks. Then, the cells were washed with PBS and fixed using 4% paraformaldehyde for 20 min. After fixing, the cells were washed twice with PBS and stained with 0.5% crystal violet for 20 min. For the transwell invasion assay, the cells were seeded at a density of 2 × 10^5^ cells in the upper chamber of an 8 μm transwell insert (Corning) coated with 1 mg of matrigel (BD Bioscience) that was plated in a 24-well cell culture plate. The lower chamber was filled with DMEM supplemented with 10% FBS and 1% penicillin–streptomycinand incubated for 20 h. The cells that invaded the transwell inserts were washed with PBS and fixed using 4% paraformaldehyde for 20 min. After fixing, the cells were washed with PBS and stained with 0.5% crystal violet for 20 min. The cells that remained in the upper chamber of the transwell insert were removed with a swab and analyzed under a microscope.

### 4.7. Annexin-V Staining

For cell death analysis, we used the FITC annexin-V apoptosis detection kit (BD Bioscience) according to the manufacturer’s specifications, and the stained cells were analyzed by FACS.

### 4.8. Autophagy Using GFP-LC3

A549 cells were transfected with GFP-LC3 along with siUSP14 or IU1-47. Then, 48 h after transfection, GFP-LC3 puncta were visualized using a fluorescence microscope.

### 4.9. Statistical Analysis

All data were analyzed using SPSS 12.0K for Windows. Data are presented as mean ± standard deviation of at least three independent experiments. Between-groups comparisons were performed using Student’s *t*-tests, whereas one- or two-way analysis of variance with Dunnett’s post hoc test was used to compare multiple groups. All data are presented as the mean ± standard error of the mean. *p* < 0.01 was considered statistically significant.

## Supplementary Materials

Supplementary materials can be found at https://www.mdpi.com/1422-0067/20/21/5300/s1.

## Abbreviations

UPS	Ubiquitin–proteasome system
DUBs	Deubiquitinating enzymes
USP14	Ubiquitin-specific protease 14
