# Supplementary material for: USP14 Inhibition Regulates Tumorigenesis by Inducing Autophagy in Lung Cancer In Vitro
Source: Int J Mol Sci. 2019 Oct 24;20(21):5300. doi: 10.3390/ijms20215300 (PMC6862215; doi:10.3390/ijms20215300)
Supplement: Supplementary file 1 [file ijms-20-05300-s001.pdf]

International Journal of
Molecular Sciences

MDPI

Supplementary materials:

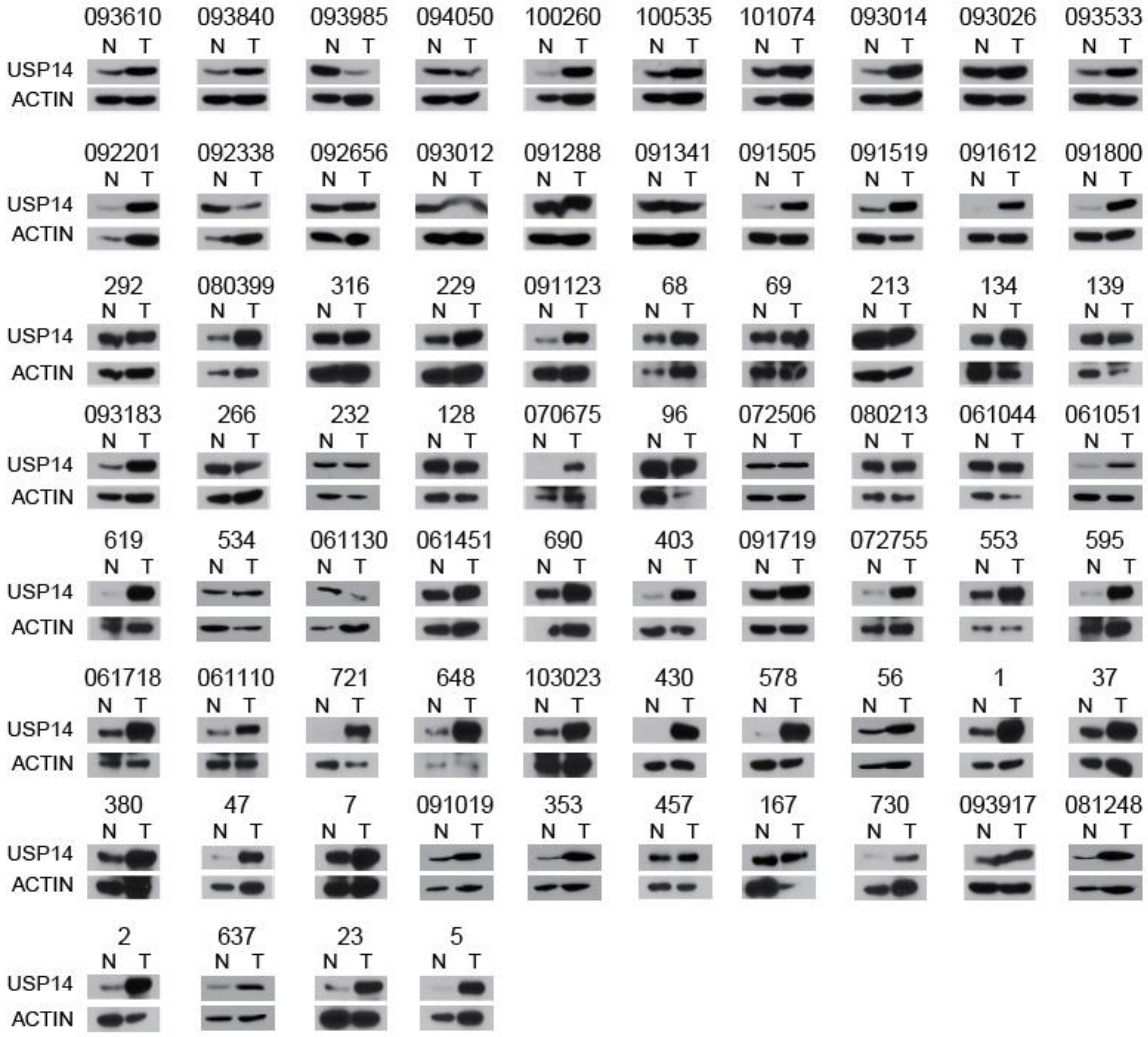

Figure S1. USP14 protein levels in 74 paired lung cancer tissues and non-tumor tissues. (N = normal lung tissue, T = lung cancer tissue).

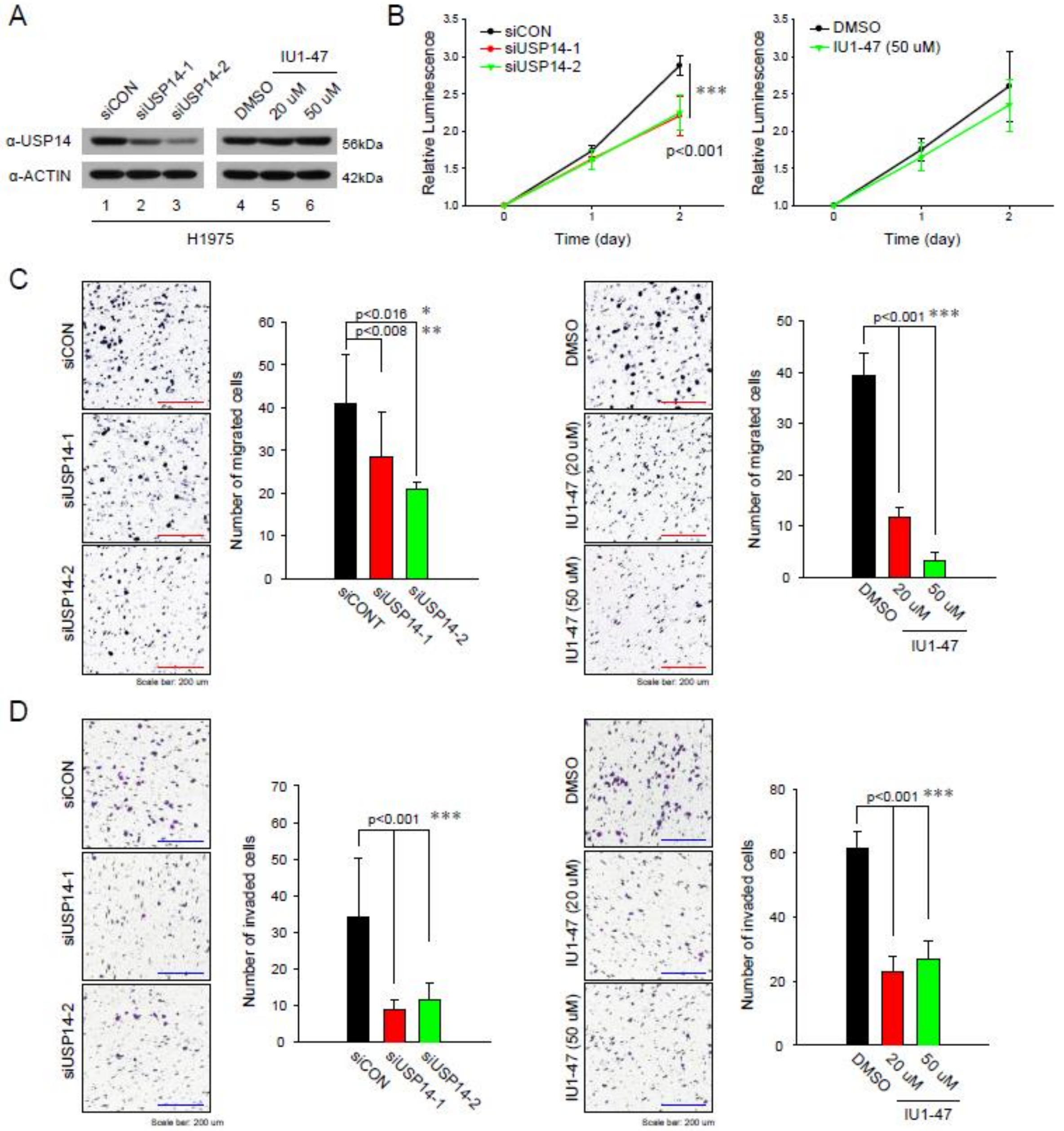

Figure S2. USP14 inhibition using siRNA and IU1-47 in H1975 lung cancer cells. (**A**) Immunoblot analysis for USP14 using siRNA and IU1-47 in H1975 cells. (**B**) Effects of USP14 knockdown or inhibition on the cell viability of H1975 (n = 5). Effects of USP14 knockdown or inhibition on cell migration (**C**) or invasion (**D**). Values are the mean ± SD of the number of invaded cells (n = 5). Scale bar = 200 μm.

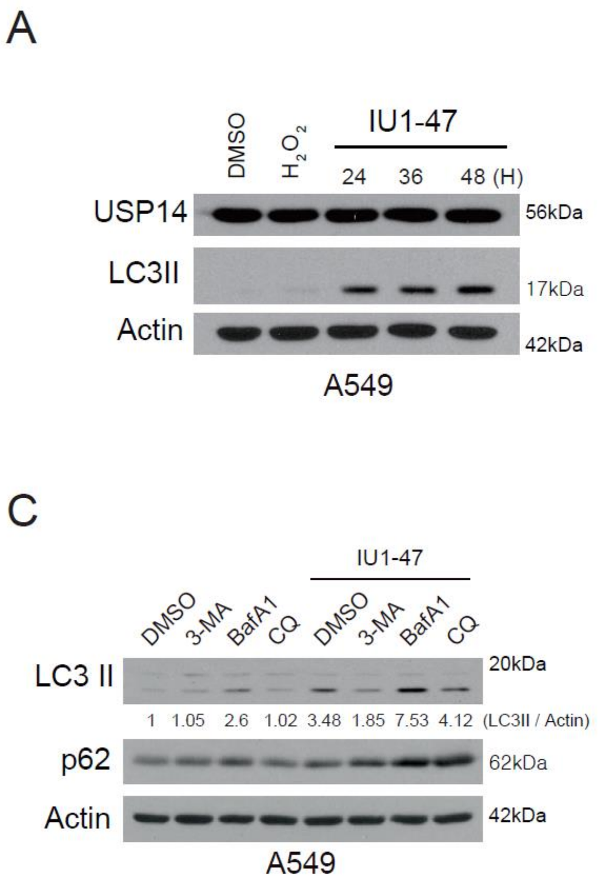

Figure S3. USP14 inhibition induces LC3 autophagy protein. (**A**–**B**) A549 cells were treated with TNF, H2O2 or USP14 inhibitor (IU1-47) and lysed and immunoblotted with anti-USP14, autophagy proteins (LC3 I, II), apoptotic cell death associated protein (PARP) and β-actin antibodies. (**C**) A549 cells were treated with DMSO, 3-Methyladenine (3-MA), Bafilomycin A1 (BafA1), chloroquine (CQ) or USP14 inhibitor (IU1-47) and lysed and immunoblotted with anti-autophagy proteins (LC3 I, II), p62 and β-actin antibodies.
